# Real-World Comparative Cost-Effectiveness Analysis of Different Classes of Disease-Modifying Therapies for Relapsing-Remitting Multiple Sclerosis in Saudi Arabia

**DOI:** 10.3390/ijerph182413261

**Published:** 2021-12-16

**Authors:** Yazed AlRuthia, Bander Balkhi, Sahar Abdullah Alkhalifah, Salman Aljarallah, Lama Almutairi, Miteb Alanazi, Abdulmalik Alajlan, Suliman M. Aldhafiri, Nuha M. Alkhawajah

**Affiliations:** 1 Department of Clinical Pharmacy, College of Pharmacy, King Saud University, P.O. Box 2454, Riyadh 11451, Saudi Arabia; bbalkhi@ksu.edu.sa (B.B.); 441202996@student.ksu.edu.sa (S.A.A.); Abdulmalikajlan@gmail.com (A.A.); Aldhafiri21@gmail.com (S.M.A.); 2 Pharmacoeconomics Research Unit, Department of Clinical Pharmacy, College of Pharmacy, King Saud University, P.O. Box 2454, Riyadh 11451, Saudi Arabia; 3 Department of Medicine, Neurology Division, College of Medicine, King Saud University, P.O. Box 3145, Riyadh 12372, Saudi Arabia; saljarallah@ksu.edu.sa (S.A.); nalkhawajah@ksu.edu.sa (N.M.A.); 4 Department of Pharmacy, King Khalid University Hospital, P.O. Box 3145, Riyadh 12372, Saudi Arabia; lamaturki.11@gmail.com (L.A.); mitalanazi@KSU.EDU.SA (M.A.)

**Keywords:** multiple sclerosis, relapsing-remitting, interferon beta-1a, natalizumab, rituximab, teriflunomide, fingolimod, cost-effectiveness, evidence-based practice

## Abstract

The very fact that multiple sclerosis (MS) is incurable and necessitates life-long care makes it one of the most burdensome illnesses. The aim of this study was to compare the cost-effectiveness of orally administered medications (e.g., fingolimod, dimethyl fumarate, and teriflunomide), interferon (IFN)-based therapy, and monoclonal antibodies (MABs) (e.g., natalizumab and rituximab) in the management of relapsing-remitting multiple sclerosis (RRMS) in Saudi Arabia using real-world data. This was a retrospective cohort study in which patients with RRMS aged ≥18 years without any other chronic health conditions with non-missing data for at least 12 months were recruited from the electronic health records of a university-affiliated tertiary care center. Multiple logistic regressions controlling for age, sex, and duration of therapy were conducted to examine the odds of disability progression, clinical relapse, MRI lesions, and composite outcome (e.g., relapse, lesion development on MRI, disability progression). The number of patients who met the inclusion criteria and were included in the analysis was 146. Most of the patients were female (70.51%) and young (e.g., ≤35 years of age). There were 40 patients on the orally administered agents (e.g., dimethyl fumarate, teriflunomide, fingolimod), 66 patients were on IFN-based therapy (e.g., Rebif^®^), and 40 patients were on monoclonal antibodies (e.g., rituximab and natalizumab). Patients on MABs had lower odds of the composite outcome (OR = 0.17 (95% CI: 0.068–0.428)). The use of orally administered agents was dominant (e.g., more effective and less costly), with average annual cost savings of USD −4336.65 (95% CI: −5207.89–−3903.32) and 8.11% higher rate of effectiveness (95% CI: −14.81–18.07) when compared with Rebif^®^. With regard to the use of MABs in comparison to Rebif^®^, MABs were associated with higher cost but a better rate of effectiveness, with an average additional annual cost of USD 1381.54 (95% CI: 421.31–3621.06) and 43.11% higher rate of effectiveness (95% CI: 30.38–61.15) when compared with Rebif^®^. In addition, the use of MABs was associated with higher cost but a better rate of effectiveness, with an average additional annual cost of USD 5717.88 (95% CI: 4970.75–8272.66) and 35% higher rate of effectiveness (95% CI: 10.0–42.50) when compared with orally administered agents. The use of MABs in the management of RRMS among the young patient population has shown to be the most effective therapy in comparison to both IFN-based therapy (e.g., Rebif^®^) and orally administered agents, but with higher cost. Orally administered agents resulted in better outcomes and lower costs in comparison to IFN-based therapy. Future studies should further examine the cost-effectiveness of different disease-modifying therapies for the management of RRMS using more robust study designs.

## 1. Introduction

Multiple sclerosis (MS) is a complex neurodegenerative inflammatory disease that affects the central nervous system (CNS) by demyelinating the myelin sheaths resulting in severe physical and/or cognitive disability [1,2]. The disease onset is mostly at 20 to 40 years of age [2], and its prevalence has significantly increased in low- and middle-income countries over the last decade, which puts a huge burden on the already strained healthcare budgets in these countries [3]. It is estimated that approximately 2.8 million patients worldwide have multiple sclerosis, with a prevalence rate of 35.9 cases per 100,000 people [4,5]. The incidence rate of MS among women is believed to be as high as two times that of their male counterparts [5]. The reported prevalence of MS in Saudi Arabia is estimated to be 40.4 per 100,000 people, including non-Saudis [5,6]. However, the prevalence is much higher among Saudis, with an estimated prevalence rate of 61.95 per 100,000 people based on a recently published study that used a national registry of MS in Saudi Arabia [6]. Moreover, the mean age of MS onset was 27.8 years, which is similar to the published estimates from other Middle Eastern countries [6].

There are four main types of MS: relapsing-remitting MS (RRMS), primary progressive MS (PPMS), secondary progressive MS (SPMS), and progressive-relapsing MS (PRMS), depending on the disease presentation [7]. Approximately 87% of patients with MS are diagnosed with RRMS, and their symptoms are characterized by episodes of neurological dysfunction (e.g., relapses), such as diplopia, limb weakness, neurogenic bladder, bowel dysfunction, unilateral optic neuritis, and Lhermitte’s sign (trunk and limb paresthesia evoked by neck flexion), followed by periods of complete or partial recovery of symptoms. These symptoms may persist, leading to a progressive phase of the disease one to two decades after the onset [7]. Although the exact etiology of MS is still unknown, there are some risk factors that may provoke MS, including genetic and environmental factors, infections (particularly Epstein-Barr virus), low levels of vitamin D, and cigarette smoking [8]. The diagnosis of MS is based on the McDonald criteria, which uses a combination of clinical examination of episodes of neurologic impairment and magnetic resonance imaging (MRI) [9]. In addition, lumbar punctures (LP) for cerebrospinal fluid (CSF) analysis, evoked potentials, and blood samples analysis may be diagnostically helpful [1].

MS can be treated with different disease-modifying therapies to improve the quality of life and minimize the relapses among patients with relapsing-remitting MS, however, these therapies have no significant benefit in progressive MS in which the neurological disability continues to worsen over time [10]. In the early 1990s, the first immunosuppressive disease-modifying therapy (DMT) was the subcutaneous interferon (IFN) β-1b, which was approved for the treatment of MS [11]. Interferons and other DMTs demonstrated a positive impact in reducing the annual relapse rate by ≥30% in comparison to placebo [10]. Until 2010, all the available (approved and off-label) DMTs (e.g., interferon beta-1b (Betaferon^®^), interferon beta-1a (Avonex^®^), interferon beta-1a (Rebif^®^), glatiramer acetate, rituximab, and natalizumab) for MS were administered parenterally [10,12]. However, multiple oral (e.g., fingolimod, siponimod, ozanimod, dimethyl fumarate, teriflunomide, and cladribine) and injectable medications (e.g., ocrelizumab, ofatumumab, and alemtuzumab) have been approved for the treatment of MS over the last decade [13].

These medications have variable efficacy rates in preventing clinical relapses and radiological changes [13]. For example, some studies have found no significant difference in the rates of relapse and/or disability progression between Interferon beta-1b (Betaferon^®^) and Interferon beta-1a (Avonex^®^ and Rebif^®^). However, in another study that examined the relative clinical efficacy rates of Rebif^®^, Betaferon^®^, and Avonex^®^ among a group of 90 patients with RRMS who were randomized to one of the three treatments and followed up for 24 months, patients on Rebif^®^ had the lowest relapse rates in comparison to their counterparts on Betaferon^®^ and Avonex^®^ [14]. Although rituximab has not been approved yet by the United States Food and Drug Administration (USFDA) for the treatment of MS, it has been used extensively as an off-label medication to manage MS. Moreover, it has shown superior efficacy in managing RRMS in comparison to fingolimod, and has a better safety profile than natalizumab [15,16]. Using the Swedish national registry for MS, rituximab was found to be superior in reducing the annualized relapse rate and resulted in 87% and 85% reductions in the rates of relapse and discontinuation, respectively, in comparison to interferon beta and glatiramer acetate [17]. In addition, using the clinician-reported outcomes, such as the clinical relapse and radiological changes on MRI, for MS patients on rituximab, natalizumab, fingolimod, or dimethyl fumarate at the Rocky Mountain MS Center at the University of Colorado in the United States, the odds of experiencing disease activity after 24 months of follow-up were higher for patients on fingolimod and those on dimethyl fumarate in comparison to rituximab. However, no significant difference in the odds of experiencing disease activity was noticed for patients on natalizumab in comparison to their counterparts on rituximab [18]. Nonetheless, the use of rituximab, natalizumab, ocrelizumab, interferons, or other injectable DMTs in the management of MS is associated with higher rates of nonadherence, particularly among patients with chronic health conditions [19,20]. Therefore, oral DMTs are believed to have better adherence rates among MS patients [19]. However, oral DMTs can cause serious adverse events, such as fetal abnormalities with fingolimod, hepatotoxicity with teriflunomide, and lymphopenia with cladribine [21]. Furthermore, oral DMTs, such as teriflunomide, were found to be less effective in managing MS in comparison to injectable DMTs, such as natalizumab, based on the findings of a single-centered retrospective cohort study that was conducted at the University of Washington in St. Louis, United States [22]. Although the annualized relapse rates were slightly lower with fingolimod compared with dimethyl fumarate and teriflunomide, no significant difference was found between the three medications in the disability outcomes over 2.5 years of follow-up using the global MSBase cohort study database [23].

It is estimated that the direct and indirect costs for MS in the United States ranged from $8528 to $54,244 per patient per year, and MS is ranked second behind heart failure in terms of the total direct healthcare costs [24]. Although many studies have evaluated the cost-effectiveness of different oral and injectable DMTs, most of these studies used hypothetical cohorts and depended on assumptions and inputs from published randomized controlled trials and observational studies with high levels of variability [25,26,27]. In a cohort simulation model that evaluated the cost-effectiveness of oral DMTs versus interferon beta-1a (e.g., Avonex^®^ and Rebif^®^) in the management of RRMS in Saudi Arabia, Rebif^®^ was found to be the optimal therapy using a cost-effectiveness threshold of USD 100,000 per quality adjust life year (QALY). However, the model inputs were taken from the published literature, and the different DMTs were not evaluated using real-world data [28]. Therefore, we aimed to examine the relative clinical effectiveness rates and costs of different oral and injectable DMTs for the management of RRMS using real-world data from Saudi Arabia due to the rising incidence and prevalence rates of MS in the kingdom.

## 2. Methods

### 2.1. Study Design and Population

This was a single-centered retrospective chart review study of patients with relapsing-remitting multiple sclerosis (RRMS). Patients with RRMS were recruited from the outpatient neurology clinics at King Saud University Medical City (KSUMC), which is one of the largest and well-known tertiary healthcare institutions in the kingdom and is affiliated with King Saud University in Riyadh, Saudi Arabia. The Cerner electronic medical record (EMR) was launched in KSUMC back in May 2015. Therefore, only patients who were diagnosed in May 2015 or after were recruited. Patients who were diagnosed before May 2015, as well as those with missing observations, were excluded from the study. Additionally, patients who have been diagnosed and treated for less than a year and those with other types of MS, such as secondary progressive MS (SPMS) and primary progressive MS (PPMS), were excluded.

### 2.2. Ethical Approval of the Study

No personal identifiers, such as name or national ID, were collected and the study adhered to the ethical principles of the Helsinki declaration [29]. The study protocol was approved by the research ethics committee of the College of Medicine at King Saud University, Riyadh, Saudi Arabia (project no. E-19-4512).

### 2.3. Data Collection and Study Variables

Three individuals were involved in the data collection process (e.g., one pharmacist and two pharmacy interns). Age, sex, medical characteristics (e.g., chronic health conditions and prescription medications), type and medication used for MS treatment, such as interferon-based therapy (e.g., interferon beta-1a or interferon beta-1b), oral agents (e.g., teriflunomide, dimethyl fumarate, fingolimod), or monoclonal antibodies (MABs) (e.g., natalizumab, alemtuzumab, rituximab), duration of therapy, rate of admission or emergency department visits due to MS, the ordered laboratory tests and their annual frequencies, and imaging studies (e.g., magnetic resonance imaging (MRI), ultrasound, computed tomography (CT)) and their annual frequencies, and clinic visits were collected. The primary outcome of this study was a composite of lesions on MRI, clinical relapses, and disability progression as assessed by the treating neurologist. Costs of lab tests, imaging studies, clinic visits, and prescription drugs were retrieved from the Saudi Ministry of Health cost center.

### 2.4. Statistical Analysis

The minimum sample size of the study was estimated to be 115 patients based on the odds ratio (OR) of clinical relapse, disability progression, and/or lesions on MRI (e.g., composite outcome) of 2.43 for patients treated with interferon in comparison to their counterparts on other therapies, such as oral agents, α = 0.05, β = 0.05, and power of 0.95 [30]. Patients’ baseline characteristics were described using frequencies and percentages. In order to explore the odds of clinical relapse, disability progression, lesions on MRI, and the composite outcome for patients on oral agents, interferon-based therapy, and MABs, four multiple logistic regressions were conducted controlling for age, sex, and duration of therapy. The incremental cost-effectiveness ratio (ICER) was calculated based on the mean difference in the annual total cost in USD ($) and rate of composite outcome (e.g., reduction in the rate of disability progression, clinical relapse, and lesions on MRI). Three cost-effectiveness comparisons (e.g., MABs vs. interferon, oral agents vs. interferon, and MABs vs. oral agents) using non-parametric bootstrapping with 10,000 replications with propensity score matching based on the duration of therapy, age, and sex were conducted to generate the 95% confidence intervals (e.g., 95% CI) for the difference in the annual mean total costs and rates of the composite outcome. The statistical analyses were performed using SAS^®^ version 9.4 (SAS^®^ Institute Inc., Cary, NC, USA).

## 3. Results

**Figure 1:**
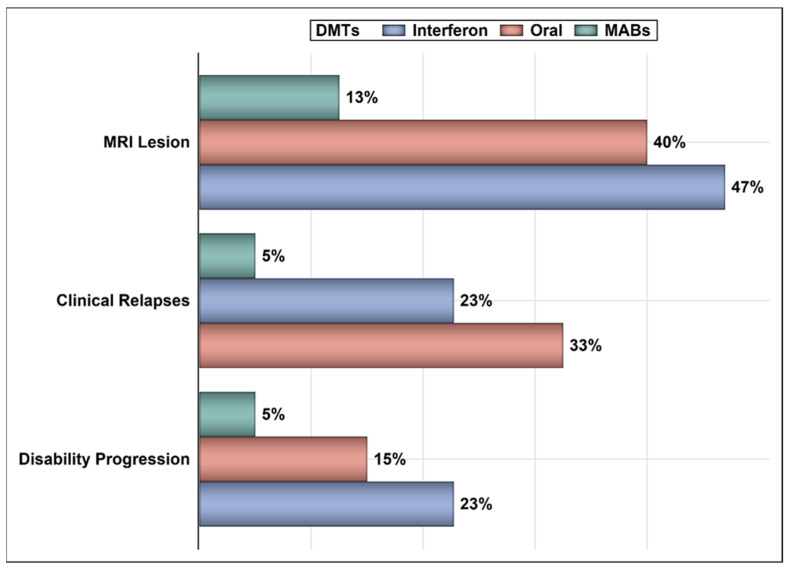
The percentages of patients on oral agents, interferon, and MABs who had disability progression, clinical relapse, and lesions on MRI.

Out of 199 patients with RRMS whose EMRs were reviewed, 146 patients (73.37%) met the inclusion criteria and were included in the analysis. Most of the patients were female (65.07%) and aged ≤ 35 years (71.24%). About 27% of the patients were treated with oral agents (e.g., dimethyl fumarate, teriflunomide, and fingolimod), 45.21% were treated with interferon beta-1a (e.g., Rebif^®^), and 27.39% were treated with MABs (e.g., natalizumab and rituximab). The mean age of patients who were treated with oral agents, MABs, and interferon beta-1a were 33.66 years, 29.87 years, and 30.97 years, respectively, with no statistically significant difference between the three treatment groups (*p* > 0.05). Approximately 53% of the patients have been treated for one to two years (Table 1). The number of patients who experienced the composite outcome was 68 (46.58%). About 21% of patients had a clinical relapse, 15.75% had disability progression, and 35.62% had lesions on MRI. The rate of clinical relapse, disability progression, and lesions on MRI across the treatment groups (e.g., oral agents, interferon, and MABs) are shown in Figure 1.

Controlling for age, sex, and duration of therapy, patients on interferon had 2.83 times higher odds of having a disability progression (OR = 2.83, 95% CI = 1.089–7.366, *p*-value = 0.033) in comparison to their counterparts on oral agents or MABs. Conversely, patients on MABs had 79.9% lower odds of having a disability progression (OR = 0.201, 95% CI = 0.044–0.927, *p*-value = 0.039) in comparison to their counterparts on oral agents or interferon. Similarly, patients on MABs had 83.3% lower odds of having a clinical relapse (OR = 0.167, 95% CI = 0.037–0.753, *p*-value = 0.019) in comparison to their counterparts on oral agents or interferon. On the other hand, patients on oral agents had 2.55 times higher odds of having a clinical relapse (OR = 2.55, 95% CI = 1.019–6.407, *p*-value = 0.045) in comparison to their counterparts on MABs or interferon. The odds of having lesions on MRI were the highest among patients on interferon (OR = 3.583, 95% CI = 1.616–7.940, *p*-value = 0.001) and the lowest among those on MABs (OR = 0.201, 95% CI = 0.07–0.578, *p*-value = 0.003). Similarly, patients on interferon had the highest odds of experiencing the composite outcome (OR = 3.409, 95% CI = 1.646–7.057, *p*-value = 0.001), and those on MABs had the lowest (OR = 0.170, 95% CI = 0.068–0.428, *p*-value = 0.0002) (Table 2).

**Figure 2:**
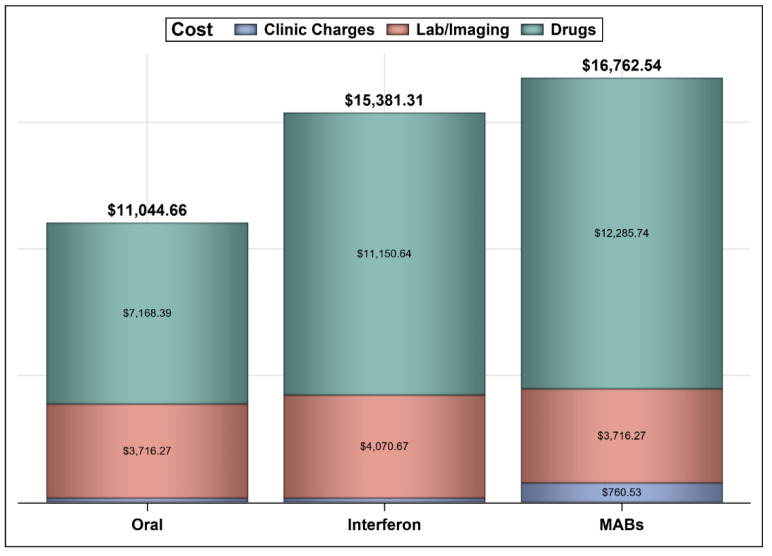
The mean annual costs of oral agents, interferon, and MABs.

The mean annual costs for oral agents, interferon, and MABs are shown in Figure 2. Comparing oral agents to interferon, oral agents would result in a mean annual cost saving of USD −4366.68 per patient (95% CI = −5207.89–−3903.32), and the mean difference in the rate of effectiveness was 8.11% (95% CI = −14.81–18.07) resulting in an incremental cost-effectiveness ratio (ICER) of USD −53,498.835 per a composite event prevention. This means that the use of oral agents, such as fingolimod and teriflunomide, instead of interferon beta-1a (e.g., Rebif^®^) achieves a better outcome at lower cost with a 96.05% confidence level based on the propensity score matching with 10,000 bootstrap replications. However, there is a small chance (e.g., 3.94%) that the use of oral agents would result in lower costs and worse outcomes. The second analysis compared MABs versus interferon and found the mean difference in the annual cost per patient to be USD 1381.54 (95% CI: 421.31–3621.06), and the mean difference in the rate of effectiveness was 43.11% (95% CI: 30.38–61.15) resulting in an ICER of USD 3204.27 per composite event prevention. This means that the use MABs, such as natalizumab and rituximab, instead of interferon beta-1a (e.g., Rebif^®^) would result in more cost and better outcomes with a 99.59% confidence level based on the propensity score matching with 10,000 bootstrap replications. The third analysis compared MABs versus oral agents and found the mean difference in the annual cost per patient to be USD 5717.88 (95% CI: 4970.75–8272.66), and the mean difference in the rate of effectiveness was 35% (95% CI: 10.0–42.50) resulting in an ICER of USD 16,336.81 per composite event prevention in 99.9% of the model runs. This means that the use of MABs, such as natalizumab and rituximab, would result in higher cost and better outcomes in comparison to oral agents (e.g., dimethyl fumarate, teriflunomide, and fingolimod) with a 99.9% confidence level based on the propensity score matching with 10,000 bootstrap replications. The mean annual costs and effectiveness rates, alongside their 95% confidence intervals for the three different comparisons, are shown in Table 3.

## 4. Discussion

Multiple sclerosis is one of the most debilitating chronic health conditions, with a rising incidence among the young populations in middle- and low-income countries [1,2,3]. In Saudi Arabia, the prevalence of MS is believed to be as high as 61.95 per 100,000 people [6]. This puts the policymakers in the public healthcare system, who are ethically responsible for promoting the welfare of patients and preserving their dignity [31], at an enormous pressure to provide care for those affected by the disease [4,24]. Although multiple prescription medications are currently available to manage MS, no study has so far investigated the clinical value of those therapies considering their cost for the management of MS in Saudi Arabia. Therefore, we aimed to explore the cost-effectiveness of different classes of therapies used in the management of RRMS, which is the most prevalent type of MS, using real-world data from the public payer’s perspective. Exploring the therapeutic value as well as the cost of MS therapies in real-world settings is important due to the fact that most cost-effectiveness analyses that were conducted before were based on trial-based efficacy estimates [32]. Orally administered agents were associated with the lowest direct medical cost (e.g., drug acquisition cost, lab, and imaging studies) in comparison to IFN-based therapy (e.g., Rebif^®^) and MABs (e.g., natalizumab and rituximab). In addition, orally administered agents resulted in better effectiveness in terms of lower rates of the composite outcome (e.g., lower rates of MRI lesions, clinical relapse, and disability progression), and were dominant in comparison to Rebif^®^ in 96.05% of the model runs. This makes the use of orally administered agents attractive since they lead to better clinical outcomes and generate cost savings in comparison to IFN-β-1b, which is in line with a Markov model-based analysis that compared the cost-effectiveness of teriflunomide versus IFN-β-1b among relapsing multiple sclerosis patients from the payer’s perspective in China using efficacy estimates from a network meta-analysis, and found teriflunomide to be dominant [33]. Additionally, oral DMTs, such as fingolimod and particularly teriflunomide, were found to be cost-effective in comparison to the other DMTs and should be used as first-line treatment for RRMS in the management RRMs from the Chinese healthcare perspective [34]. On the other hand, these findings are at odds with another literature-based Markov model analysis that evaluated the cost-effectiveness of orally administered agents in comparison to IFN from the payer’s perspective in Saudi Arabia and found Rebif^®^ to be the optimal therapy at a willingness to pay (WTP) threshold of USD 100,000 [28].

Unsurprisingly, MABs (e.g., rituximab and natalizumab) resulted in better outcomes in comparison to both Rebif^®^ and orally administered agents. The odds of clinical relapse among patients on MABs were 83.3% lower in comparison to patients on orally administered agents and Rebif^®^. In addition, the odds of disability progression and MRI lesions were 79.9% lower among patients on MABs compared to Rebif^®^ and orally administered agents. This is consistent with previously published studies that found rituximab and natalizumab to be superior in comparison to oral DMTs and IFN-based therapies in the management of RRMS [15,16,17,18]. However, this comes at a higher cost when compared to IFN-based therapies or oral DMTs. Although the mean annual incremental cost of MABs in comparison to Rebif^®^ was relatively low (e.g., USD 3204.27 per composite outcome prevention), its mean annual incremental cost was much higher in comparison to oral DMTs (e.g., USD 16,336.81 per composite outcome prevention). Nonetheless, the use of MABs was associated with better outcomes in more than 99% of the model runs. These findings are consistent with a 30-year Markov model that examined the cost-effectiveness of natalizumab versus IFN-based therapies among highly active RRMS patients using trial-based efficacy rates and was conducted from different United Kingdom (U.K.) cost perspectives [35]. The ICERs for natalizumab compared with IFN-based therapies were lower than the cost-effectiveness threshold in the U.K., which suggests that natalizumab is a cost-effective treatment for highly active RRMS [35]. In another Markov model-based study that was conducted from the Colombian healthcare system perspective, natalizumab was associated with higher QALYs compared to fingolimod and lower costs [36]. However, the model depended on utilities extracted from the literature, and resource use and costs were based on expert opinions [36]. Therefore, the model does not represent the real costs and efficacy rates seen in real-world practice. However, another Markov model-based study that used literature-based efficacy estimates and collected cost data from different clinics and neurology specialists in Fars province in Iran to examine the cost-effectiveness of fingolimod versus natalizumab from the societal perspective found fingolimod to be dominant (e.g., higher QALYs and lower costs) [37]. Therefore, the findings of previously published cost-effectiveness analyses that were based on literature-based efficacy estimates and expert opinions regarding the utilization rates of different healthcare resources are controversial and largely dependent on the model inputs, which in many cases do not represent the real-world settings [32]. Thus, the value of real-world comparative effectiveness and cost-effectiveness analyses is instrumental in shaping different treatment guidelines and policies about the management of RRMS [10,32].

Although this is the first study to the best of our knowledge that compared the effectiveness and costs of different MS DMTs using real-world data in Saudi Arabia, multiple limitations should be acknowledged. First, the data were extracted from a single center, and the recruited patients did not have other chronic health conditions. This limits the generalizability of the findings. However, few healthcare institutions in Saudi Arabia are specialized in managing MS, and KSUMC is one of the main referral hospitals. Secondly, the cost-effectiveness analysis was not based on the Extended Disability Status Scale (EDSS) or QALYs, but rather a composite outcome of MRI lesions, clinical relapse, and/or disability progression. Additionally, some patients may have received multiple restorative rehabilitation therapy, which may delay the disease progression, but this was not controlled for in the analysis due to the lack of data [38]. However, the correlation between clinical relapse, disability progression, and MRI lesions is significant, according to a meta-analysis that included 19 randomized, double-blind controlled trials of RRMS patients. Therefore, these endpoints (e.g., MRI lesions, clinical relapse, and disability progression) are considered valid surrogate endpoints of EDSS [39]. Thirdly, no sensitivity analysis of the drug acquisition costs and other healthcare resource use (e.g., clinic visits, lab, imaging, and drug administration and monitoring costs) was conducted. Nonetheless, the analysis was conducted based on real-world data and from the public healthcare payer perspective. Therefore, the ICERs will be different if the drug acquisition costs have changed or discounted. Fourthly, the study evaluated the cost-effectiveness of different classes of DMTs rather than individual agents due to its small sample size.

## 5. Conclusions

The healthcare resource utilization rates and effectiveness for different MS DMTs in real-world settings are different from the ones reported in clinical trials. Therefore, the evaluation of different MS DMTs should be further examined using real-world data. The findings of this study should highlight the value of different classes of MS DMTs in the management of RRMS. Future studies should examine the cost-effectiveness of different MS DMTs using data from multiple healthcare institutions and patient populations.

## Figures and Tables

**Table 1:** Patient baseline characteristics (*n* = 146).

Characteristic	Frequency (%)
Sex	
Male	51 (34.93)
Female	95 (65.07)
Age	
16 yrs–25 yrs	42 (28.77)
26 yrs–35 yrs	62 (42.47)
36 yrs–45 yrs	27 (18.49)
>45 yrs	15 (10.27)
Oral agents	
Dimethyl fumarate	2 (1.37)
Teriflunomide	15 (10.27)
Fingolimod	23 (15.75)
Interferon	
Rebif^®^ (interferon beta-1a)	66 (45.21)
Monoclonal antibodies (MABs)	
Natalizumab	28 (19.18)
Rituximab	12 (8.22)
Duration of therapy	
1 yr–2 yrs	77 (52.74)
2 yrs–3 yrs	31 (21.23)
3 yrs–4 yrs	38 (26.03)

**Table 2:** The odds of disability progression, clinical relapse, and lesions on magnetic resonance imaging (MRI).

Disability Progression	Odds Ratio (OR) ^†^	*p*-Value	95% Confidence Interval
Oral agents	0.872	0.803	0.299–2.543
Interferon	2.833	0.033 *	1.089–7.366
MABs	0.201	0.039 *	0.044–0.927
Clinical relapse			
Oral agents	2.555	0.045 *	1.019–6.407
Interferon	1.217	0.647	0.526–2.812
MABs	0.167	0.019 *	0.037–0.753
MRI lesions			
Oral agents	0.790	0.589	0.337–1.854
Interferon	3.583	0.001 *	1.616–7.940
MABs	0.201	0.003 *	0.070–0.578
Composite outcome ^‡^			
Oral agents	1.082	0.845	0.490–2.389
Interferon	3.409	0.001 *	1.646–7.057
MABs	0.170	0.0002 *	0.068–0.428

^†^ All odds ratios have been adjusted for age, sex, and duration of therapy. ^‡^ Composite outcome is the presence of one or more of the following: clinical relapse, disability progression, and MRI lesions. * *p*-value < 0.05.

**Table 3:** The mean effectiveness rates and costs of oral, interferon, and monoclonal antibodies-based therapies.

**Difference in Cost and Effectiveness Rate between Orally Administered Agents (e.g., Dimethyl Fumarate, Fingolimod, Teriflunomide) and Interferon Beta-1a**			
	**Oral Agents**	**Interferon**	**Mean Difference (95% Confidence Interval)**
Cost of treatment (USD), mean ± SD	11,044.66 ± 2128.26	15,381.31 ± 279.23	−4336.65 (−5207.89–−3903.32)
Effectiveness rate (%), mean ± SD	47.50 ± 50.57	39.39 ± 49.24	8.11 (−14.81–18.07)
**Difference in Cost and Efficacy Rate between MABs (e.g., Rituximab and Natalizumab) and Interferon Beta-1a**			
	**MABs**	**Interferon**	**Mean Difference (95% Confidence Interval)**
Cost of treatment (USD), mean ± SD	16,762.54 ± 5939.96	15,381.31 ± 279.23	1381.54 (421.31–3621.06)
Effectiveness rate (%), mean ± SD	82.50 ± 38.49	39.39 ± 49.24	43.11 (30.38–61.15)
**Difference in Cost and Efficacy Rate between MABs (e.g., Rituximab and Natalizumab) and Orally Administered Agents (e.g., Dimethyl Fumarate, Fingolimod, Teriflunomide)**			
	**MABs**	**Oral Agents**	**Mean Difference (95% Confidence Interval)**
Cost of treatment (USD), mean ± SD	16,762.54 ± 5939.96	11,044.66 ± 2128.26	5717.88 (4970.75–8272.66)
Effectiveness rate (%), mean ± SD	82.50 ± 38.49	47.50 ± 50.57	35 (10.0–42.50)

## Data Availability

The data are available upon reasonable request from the corresponding Author (Yazed AlRuthia).

## References

[1] Ghasemi N., Razavi S., Nikzad E. (2017). Multiple Sclerosis: Pathogenesis, Symptoms, Diagnoses and Cell-Based Therapy. Cell J..

[2] Reich D.S., Lucchinetti C.F., Calabresi P.A. (2018). Multiple Sclerosis. N. Engl. J. Med..

[3] Heydarpour P., Khoshkish S., Abtahi S., Moradi-Lakeh M., Sahraian M.A. (2015). Multiple Sclerosis Epidemiology in Middle East and North Africa: A Systematic Review and Meta-Analysis. Neuroepidemiology.

[4] Wallin M.T., Culpepper W.J., Nichols E., Bhutta Z.A., Gebrehiwot T.T., Hay S.I., Khalil I.A., Krohn K.J., Liang X., Naghavi M. (2019). Global, regional, and national burden of multiple sclerosis 1990–2016: A systematic analysis for the Global Burden of Disease Study 2016. Lancet Neurol..

[5] Walton C., King R., Rechtman L., Kaye W., Leray E., Marrie R.A., Robertson N., La Rocca N., Uitdehaag B., Van Der Mei I. (2020). Rising prevalence of multiple sclerosis worldwide: Insights from the Atlas of MS, third edition. Mult. Scler. J..

[6] AlJumah M., Bunyan R., Al Otaibi H., Al Towaijri G., Karim A., Al Malik Y., Kalakatawi M., Alrajeh S., Al Mejally M., Algahtani H. (2020). Rising prevalence of multiple sclerosis in Saudi Arabia, a descriptive study. BMC Neurol..

[7] Ford H. (2020). Clinical presentation and diagnosis of multiple sclerosis. Clin. Med..

[8] Connick P., De Angelis F., Parker R.A., Plantone D., Doshi A., John N., Stutters J., MacManus D., Carrasco F.P., Barkhof F. (2018). Multiple Sclerosis-Secondary Progressive Multi-Arm Randomisation Trial (MS-SMART): A multiarm phase IIb randomised, double-blind, placebo-controlled clinical trial comparing the efficacy of three neuroprotective drugs in secondary progressive multiple sclerosis. BMJ Open.

[9] Polman C.H., Reingold S.C., Banwell B., Clanet M., Cohen J.A., Filippi M., Fujihara K., Havrdova E., Hutchinson M., Kappos L. (2011). Diagnostic criteria for multiple sclerosis: 2010 revisions to the McDonald criteria. Ann. Neurol..

[10] Tintore M., Vidal-Jordana A., Garriga J.S. (2018). Treatment of multiple sclerosis—Success from bench to bedside. Nat. Rev. Neurol..

[11] Faissner S., Gold R. (2018). Efficacy and safety of the newer multiple sclerosis drugs approved since 2010. CNS Drugs.

[12] Alping P., Frisell T., Nováková L., Islam-Jakobsson P., Salzer J., Björck A., Axelsson M., Malmeström C., Fink K., Lycke J. (2016). Rituximab versus fingolimod after natalizumab in multiple sclerosis patients. Ann. Neurol..

[13] McGinley M.P., Goldschmidt C.H., Rae–Grant A.D. (2021). Diagnosis and treatment of multiple sclerosis: A review. JAMA.

[14] Etemadifar M., Janghorbani M., Shaygannejad V. (2006). Comparison of Betaferon, Avonex, and Rebif in treatment of relapsing–remitting multiple sclerosis. Acta Neurol. Scand..

[15] Chisari C.G., Sgarlata E., Arena S., Toscano S., Luca M., Patti F. (2021). Rituximab for the treatment of multiple sclerosis: A review. J. Neurol..

[16] Gajofatto A., Benedetti M.D. (2015). Treatment strategies for multiple sclerosis: When to start, when to change, when to stop?. World J. Clin. Cases.

[17] Spelman T., Frisell T., Piehl F., Hillert J. (2017). Comparative effectiveness of rituximab relative to IFN-β or glatiramer acetate in relapsing-remitting MS from the Swedish MS registry. Mult. Scler. J..

[18] Vollmer B.L., Nair K., Sillau S., Corboy J.R., Vollmer T., Alvarez E. (2020). Rituximab versus natalizumab, fingolimod, and dimethyl fumarate in multiple sclerosis treatment. Ann. Clin. Transl. Neurol..

[19] Bergvall N., Petrilla A.A., Karkare S.U., Lahoz R., Agashivala N., Pradhan A., Capkun G., Makin C., McGuiness C.B., Korn J.R. (2014). Persistence with and adherence to fingolimod compared with other disease-modifying therapies for the treatment of multiple sclerosis: A retrospective US claims database analysis. J. Med. Econ..

[20] Katsarava Z., Ehlken B., Limmroth V., Taipale K., Patel S.N., Niemczyk G., Rehberg-Weber K., Wernsdörfer C. (2015). Adherence and cost in multiple sclerosis patients treated with IM IFN beta-1a: Impact of the CARE patient management program. BMC Neurol..

[21] Deleu D., Mesraoua B., Canibaño B., Melikyan G., Al Hail H., El-Sheikh L., Ali M., Al Hussein H., Ibrahim F., Hanssens Y. (2018). Oral disease-modifying therapies for multiple sclerosis in the Middle Eastern and North African (MENA) region: An overview. Curr. Med. Res. Opin..

[22] Longbrake E.E., Cross A.H., Salter A. (2016). Efficacy and tolerability of oral versus injectable disease–modifying therapies for multiple sclerosis in clinical practice. Mult. Scler. J. Exp. Transl. Clin..

[23] Kalincik T., Havrdova E.K., Horakova D., Izquierdo G., Prat A., Girard M., Duquette P., Grammond P., Onofrj M., Lugaresi A. (2019). Comparison of fingolimod, dimethyl fumarate and teriflunomide for multiple sclerosis. J. Neurol. Neurosurg. Psychiatry.

[24] Adelman G., Rane S.G., Villa K.F. (2013). The cost burden of multiple sclerosis in the United States: A systematic review of the literature. J. Med. Econ..

[25] Navarro C.E., Ordóñez–Callamand E., Alzate J.P. (2020). Disease modifying therapies in multiple sclerosis: Cost–effectiveness systematic review. Farm. Hosp..

[26] Noyes K., Bajorska A., Chappel A., Schwid S.R., Mehta L.R., Weinstock-Guttman B., Holloway R.G., Dick A.W. (2011). Cost–effectiveness of disease–modifying therapy for multiple sclerosis: A population–based study. Neurology.

[27] Bozkaya D., Livingston T., Migliaccio-Walle K., Odom T. (2016). The cost-effectiveness of disease-modifying therapies for the treatment of relapsing-remitting multiple sclerosis. J. Med. Econ..

[28] Alsaqa’aby M.F., Vaidya V., Khreis N., Al Khairallah T., Al-Jedai A.H. (2017). Cost-effectiveness of oral agents in relapsing-remitting multiple sclerosis compared to interferon-based therapy in Saudi Arabia. Ann. Saudi Med..

[29] Association GAotWM (2014). World Medical Association Declaration of Helsinki: Ethical principles for medical research involving human subjects. J. Am. Coll. Dent..

[30] Bergvall N., Makin C., Lahoz R., Agashivala N., Pradhan A., Capkun G., Petrilla A.A., Karkare S.U., McGuiness C.B., Korn J.R. (2014). Relapse rates in patients with multiple sclerosis switching from interferon to fingolimod or glatiramer acetate: A US claims database study. PLoS ONE.

[31] Kooli C. (2021). COVID–19: Public health issues and ethical dilemmas. Ethics Med. Public Health.

[32] Brandes D.W., Raimundo K., Agashivala N., Kim E. (2013). Implications of Real-world Adherence on Cost-effectiveness Analysis in Multiple Sclerosis. J. Med. Econ..

[33] Xu Y., Mao N., Chirikov V., Du F., Yeh Y.C., Liu L., Liu R., Gao X. (2019). Cost–effectiveness of Teriflunomide Compared to Interferon Beta–1b for Relapsing Multiple Sclerosis Patients in China. Clin. Drug Investig..

[34] Dong Z., Hu Z., Zhou X., Wang J., Wang J., Wang C., Sun G., Tao Q. (2021). Cost–Effectiveness of Teriflunomide and Fingolimod in The First–Line Treatment of Relapsing–Remitting Multiple sclerosis: The Chinese Health System Perspective. BMC Ser..

[35] Gani R., Giovannoni G., Bates D., Kemball B., Hughes S., Kerrigan J. (2008). Cost-Effectiveness Analyses of Natalizumab (Tysabri^®^) Compared with Other Disease-Modifying Therapies for People with Highly Active Relapsing-Remitting Multiple Sclerosis in the UK. Pharmacoeconomics.

[36] Lasalvia P., Hernández F., Castañeda-Cardona C., Cuestas J.A., Rosselli D. (2020). Cost-Effectiveness of Natalizumab Compared with Fingolimod for Relapsing-Remitting Multiple Sclerosis Treatment in Colombia. Value Health Reg. Issues.

[37] Rezaee M., Izadi S., Keshavarz K., Borhanihaghighi A., Ravangard R. (2019). Fingolimod versus natalizumab in patients with relapsing remitting multiple sclerosis: A cost–effectiveness and cost–utility study in Iran. J. Med. Econ..

[38] Haselkorn J.K., Hughes C., Rae-Grant A., Henson L.J., Bever C.T., Lo A., Brown T.R., Kraft G.H., Getchius T., Gronseth G. (2015). Summary of comprehensive systematic review: Rehabilitation in multiple sclerosis: Report of the Guideline Development, Dissemination, and Implementation Subcommittee of the American Academy of Neurology. Neurology.

[39] Sormani M.P., Bonzano L., Roccatagliata L., Mancardi G.L., Uccelli A., Bruzzi P. (2010). Surrogate endpoints for EDSS worsening in multiple sclerosis: A meta-analytic approach. Neurology.

